# Supplementary material for: Patterns of Intron Gain and Loss in Fungi
Source: PLoS Biol. 2004 Nov 30;2(12):e422. doi: 10.1371/journal.pbio.0020422 (PMC532390; doi:10.1371/journal.pbio.0020422)
Supplement: Table S1 — Also available at http://genes.mit.edu/NielsenEtAl/. (4.3 MB ZIP). [file pbio.0020422.st001.zip › NielsenEtAl/html/1353.html]

AN5517.1.NCU05939.1.MG08345.1.FG02237.1


```
 CLUSTAL W (1.82) Multiple Sequence Alignments - Introns Inserted


Sequence 1: NCU05939.1	1029 aa
Sequence 2: FG02237.1	1030 aa
Sequence 3: MG08345.1	1033 aa
Sequence 4: AN5517.1	1038 aa
Alignment Length: 1086 aa
Number Identitical Residues: 384 aa
Alignment Score (without introns) 18902


MG08345.1 	MASVSPPPIQQTTDVGRPRSKQQMSVNYDEAENGPVGQRSSRLQFELAQCVETTTVTTTK
NCU05939.1	--MSSPTPTQKTIADRPPRSKQRVSMTWAEEDDP---SSKTRTLTLSEQYVETKTVTTTT
FG02237.1 	--MTPSPATSQALEPRHSKPSQTVSVNISEGSQS---QRSRRFQLEVSECVETKTVTTTT
AN5517.1  	-------------------------MDDSEAQAT---ASGSIGQFSFAPATQTTVVTTTT
          	                         :   * .                  .:*..****.

MG08345.1 	TTKLAIPPLLLREPRPLSSLDTKEYPLANRPTPPELAKFTMDIEGYQQGLFPVDSSFEEA
NCU05939.1	TTQRRFSPIQIREPQ---MLDPKEYPLAAQPTPMFLKNFSSTYTVPQGFQHKGQIWS-PV
FG02237.1 	RLTRKFPHVFVRDPVPLENLDTKEYPLAMKPTPPELIEFS--YKLPGALETDDEGEDEPT
AN5517.1  	TTTTTFPPLFIKPPRAVRELDPKLYPLASSATPSALKNIRFEVGGKSVVFNEPDDTTATI
          	     :. : :: * .   **.* ****  .**  * ::              :      

MG08345.1 	MY~K0REHEPRPCSLD-VDALETPDTPQQESPTPTFFNNKPRPTRRASGIPSRNASKPYG
NCU05939.1	GG~F~GVDGNQHRVEN-IDYIEEPTVESGGSLTPSLIQRRPQPKRLQRPSSRFDSPNNSP
FG02237.1 	ET~Q0MTYSNGHSTPG-RIKAESPQESNTLVRVPKHRSQSPSEGYPRRSRASRASPTDSP
AN5517.1  	EA0I~HERNDVLRASNGVIRSVTSFNSEDNQSLSRFAASRVSNHPSPVPTSSKRRPLPLA
          	               ..      .   .     .                .    .    

MG08345.1 	SIGREEFSRSN-RPIMTSTASERMRRPFTEQRRME-----SDSAAGYLATPDATELVGGS
NCU05939.1	------LPRSMARLGVLTSDT-ELRRALAKGPRHQ---RSPRTDTDYLATPDEAERD-SG
FG02237.1 	ESSHTSNPRRLPRSNPLNAVSDKLRRSIAHGSSRREGERLSRAPSGFLATPDTSEVVGSS
AN5517.1  	DSWQGHSKTQSTRIPSEPLVCSRSSGSTSSHPRAAGLATPETESSSFSSQDSTTSRRRIR
          	.          .*        ..   . :               :.: :  . :.     

MG08345.1 	RRRSSRREPLDQ--GESAESSTQQTSLFDAASPAESE-----------ANTAYSGSVTTP
NCU05939.1	D--FDHDAPALQLVNTTEAETSQSTAYDDAESQAGSE-----------TQTVFSA-VATP
FG02237.1 	DRPARRRSSHHERQHSPEASSSAVSPHYEATSPVESD-----------SHTVFSSNVATP
AN5517.1  	SANFPRKETLLRSPLSSEVESQGDRPNLHARKDPSRSRTLVSERNRHPSRPSVSDSAESA
          	     :  .  .    .  .:    .  .* .    . :  :. .  .:..  * .. :.

MG08345.1 	PVMEADSVSLADADE---QPARAFHR------PRPTVDTIAAQDASLPSPGLSPTLQAAQ
NCU05939.1	PITDVEFQPAADYDEPLQQTVRGLH-------ASHAISAVTAQDASLPSPRLSPTLAAAQ
FG02237.1 	PITDADAEPFADIDGSLQQPLRPFN-------PRPSIDVVAAQDASLPSPRLSPTLAAAQ
AN5517.1  	IHEDQDLEVTQSQDTLFNKDVLSQNRSIDSASQLTLVDNVLAQDMCLPSPSLSPVAAMNA
          	   : :     . *   .:     : : .:::    :. : *** .**** ***.     

MG08345.1 	LQNGQQDDGDVDDDDQMEFSQQLTRQRRSRFLGNNSPANED-----DSSDVAEHALERRR
NCU05939.1	LQLGPSDD-EGADDEVTASTNAFGRSGPSQWLDETQGTGDADAEMVDDSLVLSPGRCDST
FG02237.1 	LHTADAED-EGQSSFQTSN------SDPSRWVDESQLTEDD-----TSTALVPAGHSQDD
AN5517.1  	MHAESSFDSAEDPEDETDSSFEQVDSQLSESLSRKALTAPVP---------APAGKNFEN
          	::     *.    .     :     .  *. :...  :                .     

MG08345.1 	RGGTERFSRQS--ILDTQYMVEQFDTLPNELKTLMMYQLLRRCPRKTLHMVADVVNPALK
NCU05939.1	ISPSQLDGAYHHTLMNPDMMIDSFDAMPTQMKSWMIYQLLRRCPRKTLQAVADVVNPALK
FG02237.1 	YALARPDGSPQ--VVDTQTLLEAFDSMKTEMKTFMMYQFLRRCPRPTLRVVANAVNPALK
AN5517.1  	VTSNTRVPAAS-SLMDIPNVVSFFDSMPEEMKTYLMYQLLKRCPKPTLHFVADVVNPALK
          	            ::::   ::. **::  ::*: ::**:*:***: **: **:.******

MG08345.1 	CDFIKQLPAELSLQVLSYLDYRDLCRAAQVSKLWRSIVDGNETGWKELCDRNGFELSESE
NCU05939.1	CDFFRRLPTELSLHILSYLDHRDLCRAAQVSKQWRNIVDSNETGWKELFDRDGFQLAPGE
FG02237.1 	CDFLRQLPLELGYSVLSHLDHQDLCRAAQVSKHWRNLVDRNETGWKELFDRDGYRLPPGE
AN5517.1  	CDFLTLLPPELSLNIVKYFDVQTMCRAAQVSKKWRHIINSDEKSWKELLDRDGFTLPEGE
          	***:  ** **.  ::.::* : :******** ** ::: :*..**** **:*: *. .*

MG08345.1 	LEKAISQGWGWQDPAGIYGAELDLSLQSRLTA-TEPELVKTAKPNASQ---------RTR
NCU05939.1	LQKAIAQGWGWQDPVGALEGEVDLSQHSKLTS-SESELFKSAVKSEKSDKADTSPTPRTR
FG02237.1 	LKKAIHQGWGWQDPVGANGYEKDLSMRSRLTS-TEHELTRNLRQETAS---------KSR
AN5517.1  	LQRAIREGWGWQFPNGLEDYEKDLSLSSSISEPSGSALSPLAGPSDRS-------TAVNR
          	*::** :***** * *    * ***  * :: .:   *      .  .       :. .*

MG08345.1 	PAKRKRPMAGYPSADRSKRRANTQDVHKSDG--MEPGHHHQNKEGPIAAATAAAMAVSDP
NCU05939.1	SSKRKRHAG-HTNTERSKRRVSFQDFRER-------FHQTHRSEGPMSAASAAALAVPDP
FG02237.1 	ASKRKRALNTYSAAERSKRRASAQEVVTREESKSQPEVRQHPSEGPLSAASAALNAVPDP
AN5517.1  	RPKRKANTR-VSSRKLAKRKISSSGTDQSDS--SDWKKDVSVAEGPYAAANAAAAAVPYP
          	 .***      .  . :**: . .     .    .        *** :**.**  **. *

MG08345.1 	GIGLPSLRRLHLFKSLFRRHYMIRQNWTSGKVKPGHVAFAAHPRHVITCLQFDEDKIITG
NCU05939.1	QIGLPSLRRLHLYKSLYRRHYMIRQNWTSGKVKPSHVAFAAHPRHVITCLQFDEDKIITG
FG02237.1 	QLGLPSLREMHLFKSLYRRHHMIRKSWTSGEVKPGHVAFAAHPRHVITCLQFDEDKIITG
AN5517.1  	DIGLPSLRGLHLYKSLYQRHYCMRKGWMKPGVKPRHIAFRAHDRHVVTCLQFDTDKILTG
          	 :****** :**:***::**: :*:.* .  *** *:** ** ***:****** ***:**

MG08345.1 	SDDQLIHVYDTNTGKLRQKLEGHEGGVWALQYEGNTLVSGSTDRSVRVWDIEKGLCTQVF
NCU05939.1	SDDTLIHVYDTKTGKLRTKLEGHEGGVWALQYVGNTLVSGSTDRSVRVWDIQKGICTQTF
FG02237.1 	SDDTLIHIYDTKTGKLRKKLEGHEGGVWALQYEGNMLVSGSTDRSVRVWDIERGLCQQVF
AN5517.1  	SDDTNINVYDTKTGVLRATLEGHEGGVWALEYYGNTLVSGSTDRSVRVWDIERARCTQIF
          	***  *::***:** ** .***********:* ** ***************::. * * *

MG08345.1 	YGHTSTVRCLQILMPTEVGSDTEGRPIMSPPKPLIITGSRDSQLRVWRLPEAGSRRYIQT
NCU05939.1	YGHTSTVRCLQILMPTETGEVKNGEPVMMPEKPLIITGSRDSQLRVWRLPEPGSRRYIMN
FG02237.1 	YGHTSTVRCLQILMPTETGRDSGGQAIMQPEKPLIITGSRDSQLRVWRLPEVGSRRYIQT
AN5517.1  	HGHTSTVRCLQIVLPVEVGKKADGTSDMMPKEPLIITGSRDSNLRVWKLPKPGDPGYYQS
          	:***********::*.*.*    * . * * :**********:****:**: *.  *  .

MG08345.1 	GPPANDAECPYFLRILQGHTHSVRAISAHADTLVSGSYDCSVRVWRISTGEQLHWLQGHS
NCU05939.1	GPAANEENCPYFVRILSGHAHSVRSISAHGDTLVSGSYDSTVRVWRISTGQQLHVLSGHN
FG02237.1 	GPPAHESDCPYFIRVLAGHTHSVRAISAHGDTLVSGSYDSTVRVWRISTGEALHVLHGHL
AN5517.1  	GPHVDDTDCPYFVRVLTGHQHSVRAIAAHGDTLVSGSYDCTVRVWKISTGETVHRLSGHS
          	** ..: :****:*:* ** ****:*:**.*********.:****:****: :* * ** 

MG08345.1 	QKVYSVVLDHKRNRCISGSMDSLVKIWDLETGACLYTLEGHSLLVGLLDLRDERLVSAAA
NCU05939.1	QKVYSVVLDHKRNRCISGSMDSFVKIWDLDTGACLYTLEGHSLLVGLLDLRDEKLVSAAA
FG02237.1 	QKVYSVVLDHERNRCISGSMDSLVKIWDLATGACLYTLEGHSLLVGLLDLRDERLVSAAA
AN5517.1  	LKVYSVVLDHKRNRCISGSMDNMVKVWSLETGSILYNLEGHTSLVGLLDLKCDRLVSAAA
          	 *********:**********.:**:*.* **: **.****: *******: ::******

MG08345.1 	DSTLRIWDPENGKCKSILTAHTAAITCFQHDSRKVISGSEKTVKMWDIATGECIEDLLTD
NCU05939.1	DSTLRIWDPENGKCKHTLMAHTGAITCFQHDGRKVISGSEKTVKMWDVQTGECMQDLLTD
FG02237.1 	DSTLRIWDPENGKCRNTLMAHTGAITCFQHDGRKVISGSEKTVKMWDVRTGECVQDLLSD
AN5517.1  	DSTLRIWDPETGQCKSMLSAHTGAITCFQHDGQKVISGSDRTLKMWDIRTGECVRDLLTN
          	**********.*:*:  * ***.********.:******::*:****: ****:.***::

MG08345.1 	LSGVWQVKFDGRRCVAAVQRDGLTYIE0ILDFGAVRDGKPREELGKRIVLNSS--EVQRL
NCU05939.1	LSGVWQVKFDERRCVAAVQRGNLTYVE0ILDFGAVRDGAPPEELGKRKLLNEA--EVQQL
FG02237.1 	LSGVWQVKFDERRCVAAVQRDQLTYVE0ILDFGAVRDGKPPEELGKRILLNEP--EVRAM
AN5517.1  	LGGVWQIKFNERRCVAAVQRDGMTYIE0VLDFGASRDGYSEDQLGRRIVVNRSGQEVSDS
          	*.****:**: *********. :**:* :***** *** . ::**:* ::* ...**   

MG08345.1 	LEEVEA---
NCU05939.1	LLAEGE---
FG02237.1 	IEEEA----
AN5517.1  	DEDYDLSDA
          	      :.:
```
